# Gut Dysbiosis and Intestinal Barrier Dysfunction: Potential Explanation for Early-Onset Colorectal Cancer

**DOI:** 10.3389/fcimb.2021.744606

**Published:** 2021-12-13

**Authors:** Siti Maryam Ahmad Kendong, Raja Affendi Raja Ali, Khairul Najmi Muhammad Nawawi, Hajar Fauzan Ahmad, Norfilza Mohd Mokhtar

**Affiliations:** ^1^ Department of Physiology, Faculty of Medicine, Universiti Kebangsaan Malaysia, Kuala Lumpur, Malaysia; ^2^ Department of Basic Medical Sciences, Faculty of Medicine and Health Sciences, Universiti Malaysia Sarawak, Sarawak, Malaysia; ^3^ Gastroenterology Unit, Department of Medicine, Faculty of Medicine, Universiti Kebangsaan Malaysia, Kuala Lumpur, Malaysia; ^4^ GUT Research Group, Faculty of Medicine, Universiti Kebangsaan Malaysia, Kuala Lumpur, Malaysia; ^5^ Department of Industrial Biotechnology, Faculty of Industrial Sciences and Technology, Universiti Malaysia Pahang, Gambang, Malaysia; ^6^ Center for Research in Advanced Tropical Bioscience, Universiti Malaysia Pahang, Gambang, Malaysia

**Keywords:** early onset, colorectal cancer, microbiota, tight junction proteins, host microbial interaction

## Abstract

Colorectal cancer (CRC) is a heterogeneous disease that commonly affects individuals aged more than 50 years old globally. Regular colorectal screening, which is recommended for individuals aged 50 and above, has decreased the number of cancer death toll over the years. However, CRC incidence has increased among younger population (below 50 years old). Environmental factors, such as smoking, dietary factor, urbanization, sedentary lifestyle, and obesity, may contribute to the rising trend of early-onset colorectal cancer (EOCRC) because of the lack of genetic susceptibility. Research has focused on the role of gut microbiota and its interaction with epithelial barrier genes in sporadic CRC. Population with increased consumption of grain and vegetables showed high abundance of *Prevotella*, which reduces the risk of CRC. Microbes, such as *Fusobacterium nucleatum*, *Bacteroides fragilis* and *Escherichia coli* deteriorate in the intestinal barrier, which leads to the infiltration of inflammatory mediators and chemokines. Gut dysbiosis may also occur following inflammation as clearly observed in animal model. Both gut dysbiosis pre- or post-inflammatory process may cause major alteration in the morphology and functional properties of the gut tissue and explain the pathological outcome of EOCRC. The precise mechanism of disease progression from an early stage until cancer establishment is not fully understood. We hypothesized that gut dysbiosis, which may be influenced by environmental factors, may induce changes in the genome, metabolome, and immunome that could destruct the intestinal barrier function. Also, the possible underlying inflammation may give impact microbial community leading to disruption of physical and functional role of intestinal barrier. This review explains the potential role of the interaction among host factors, gut microenvironment, and gut microbiota, which may provide an answer to EOCRC.

## Introduction

The rising prevalence of early-onset colorectal cancer (EOCRC) has fascinated researchers worldwide for more than 10 years (Siegel et al., 2009). A comprehensive population-based study of seven high-income countries gave prominent examples of the increasing incidence of EOCRC (Araghi et al., 2019). The study reported a decline or stabilization of the incidence of late-onset CRC in Australia, Canada, Denmark, Ireland, New Zealand, Norway, and the United Kingdom (Araghi et al., 2019). Nonetheless, colon cancer incidence has increased in patients aged 0–49 years in Denmark, New Zealand, Australia, and the United Kingdom. A similar trend was noticed in rectal cancer, in which the incidence has remarkably increased in comparable age groups in Canada, Australia, and the United Kingdom (Araghi et al., 2019). In the United States, the mortality rate of rectal cancer in patients aged less than 55 years has increased by 1% (Ahnen et al., 2014; Siegel et al., 2020). In Asian population, data from Korea, Taiwan, and Japan show similar rising trend both genders except in Hong Kong, where rectal cancer is more prominent in males than in females (Sung et al., 2019). Therefore, the recommended age for CRC screening was reviewed and changed to less than 40 years of age. The age for screening must be regularly updated based on the current incidence and prevalence of specific countries to make certain of the threshold age for screening (Siegel et al., 2020).

The clinicopathological presentation of EOCRC was reported to be different than that of late-onset CRC. The anatomical sites of EOCRC are more in the distal colon and rectum with histopathological features of mucinous and signet ring and poorly differentiated appearance (You et al., 2012; Ahnen et al., 2014; Zhang et al., 2020). EOCRC has no exclusive risk factor (Song and Chan, 2019; Siegel et al., 2020). Available evidence on potential EOCRC risk factors, including obesity, alcohol consumption, cigarette smoking, and diabetes mellitus, is conflicting. The systematic review and meta-analysis conducted by O’Sullivan et al. (2021) revealed an increased risk of EOCRC in patients with obesity (pooled relative risk [RR]: 1.54 [1.01–2.35]) and alcohol consumption (pooled RR: 1.71 [1.62–1.80]) but not in patients with cigarette smoking (pooled RR: 1.35 [0.81–2.25]). A more recent population-based case–control study of 175 patients with EOCRC and 253 healthy controls failed to show any risk association of EOCRC with obesity (odds ratio [OR]: 0.59 [0.34–1.01]), alcohol consumption (OR: 1.07 [0.49–2.32]), cigarette smoking (OR: 0.79 [0.40–1.57]), and diabetes mellitus (OR: 1.75 [0.57–5.32]) (Chang et al., 2021). Some dietary habits, such as low intake of fruits, vegetables, and fibers and high intake of red meat and processed meat, are associated with late-onset CRC but are not well established in EOCRC. **Tables 1** and **2** summarize the recent evidence regarding the potential risk factors for EOCRC.

**Table 1:** Recent published studies on the potential risk factors for EOCRC.

Potential Risk Factors	Study	Country	Study Design	Participants	Comments (positive association)	Comments (no or inverse association)
**Obesity**	Chang et al. (2021)	Canada	Population-based case control study	175 EOCRC		OR for EOCRC in:
				253 controls		Protective effect:
						a) overweight: 0.57 (0.34-0.94)
						No association:
						b) obese: 0.59 (0.34-1.01)
	O’Sullivan et al. (2021)	–	Systematic review & meta-analysis	7 studies	Pooled RR for EOCRC: 1.54 (1.01-2.35)	OR for EOCRC in:
	Low et al. (2020)	USA	Case-control study of US Veterans who underwent colonoscopy	651 EOCRC		
				67,416 controls		Protective effect:
						a) overweight: 0.69 (0.56-0.87) b) obese: 0.69 (0.55-0.86)
	Gausman V et al. (2019)	USA	Retrospective, single centre cohort	269 EOCRC		No association:
				2,802 late-onset CRC		Compared with controls, OR: 0.98 (0.95-1.00)
				1,122 controls		Compared with late-onset CRC, OR: 0.98 (0.95-0.99)
	Glover et al. (2019)	USA	Population-based cohort analysis (National Database- Explorys)	Definition of EOCRC: 20-39 years of age	Compared with no CRC, OR for EOCRC: 1.82 (1.62-2.04)	No association:
				EOCRC rate: 18.9/100,000		Compared with late-onset CRC, OR for EOCRC: 0.7 (0.62-0.8)
	Liu et al. (2019)	USA	Prospective cohort study (The Nurses’ Health Study II)	85,256 women free of cancer and inflammatory bowel disease at enrolment 114 EOCRC	Compared with women with normal BMI, RR for EOCRC:	
					a) BMI 25-29.9: 1.37 (0.81-2.30) b) BMI >30: 1.93 (1.15-3.25)	
					compared with women with weight gain <5kg, RR for EOCRC:	
					a) weight gain 20-39.9kg: 1.65 (0.96-2.81) b) weight gain >40kg: 2.15 (1.01-4.55)	
	Syed et al. (2019)	USA	Population-based cohort analysis (National Database- Explorys)	68,860 total CRC cases.	Compared with late-onset CRC, OR for EOCRC: 1.14 (1.08-1.20)	
				5,710 EOCRC	Compared with control group, OR for EOCRC: 2.88 (2.74-3.04)	
**Alcohol consumption**	Archambault et al. (2021)	–	Meta-analysis of 13 population-based studies.	3,767 EOCRC	Pooled OR (heavy alcohol consumption): 1.25 (1.04-1.50)	Pooled OR (alcohol abstinence): 1.23 (1.08-1.39)
				4,049 controls		
				23,437 late-onset CRC 35,311 older controls		
	Chang et al. (2021)	Canada	Population-based case control study	175 EOCRC		No association:
				253 controls		OR for EOCRC in daily consumption: 1.07 (0.49-2.32)
	O’Sullivan et al. (2021)		Systematic review & meta-analysis	3 studies	Pooled RR for EOCRC: 1.71 (1.62-1.80)	
	Glover et al. (2019)	USA	Population-based cohort analysis (National Database- Explorys)	Definition of EOCRC: 20-39 years of age		No association:
				EOCRC rate: 18.9/100,000		Compared with no CRC, OR for EOCRC: 0.91 (0.66-1.25)
						Protective effect:
						Compared with late-onset CRC, OR for EOCRC: 0.72 (0.53-0.99)
	Syed et al. (2019)	USA	Population-based cohort analysis (National Database- Explorys)	68,860 total CRC cases		Protective effect:
				5,710 EOCRC.		Compared with late-onset CRC, OR for EOCRC: 0.83 (0.78-0.88)
**Cigarette Smoking**	Archambault et al. (2021)	–	Meta-analysis of 13 population-based studies.	3,767 EOCRC		No association:
				4,049 controls		OR: 0.96 (0.92-1.01)
				23,437 late-onset CRC 35,311 older controls		
	Chang et al. (2021)	Canada	Population-based case control study	175 EOCRC		No association:
				253 controls		OR for EOCRC in heavy smoker: 0.79 (0.40-1.57)
	O’Sullivan et al. (2021)		Systematic review & meta-analysis	20 studies		No association:
						Pooled RR for EOCRC: 1.35 (0.81-2.25)
	Low et al. (2020)	USA	Case-control study of US Veterans who underwent colonoscopy	651 EOCRC		No association:
				67,416 controls		OR for EOCRC in current smoker: 1.10 (0.89-1.35)
	Glover et al. (2019)	USA	Population based cohort analysis (National Database- Explorys)	Definition of EOCRC: 20-39 years of age	Compared with no CRC, OR for EOCRC: 2.68 (2.41-2.97)	
				EOCRC rate: 18.9/100,000	Compared with late-onset CRC, OR for EOCRC: 1.19 (1.07-1.32)	
	Kelty et al. (2019)	Australia	Retrospective population-based cohort	713,085 EOCRC	OR: 2.02 (1.73-2.35)	
				306,329 late-onset CRC		
	Syed et al. (2019)	USA	Population-based cohort analysis (National Database- Explorys)	68,860 total CRC cases. 5,710 were EOCRC.		Protective effect:
						Compared with late-onset CRC, OR for EOCRC: 0.83 (0.79-0.88)
**Family history of cancer**	Chang et al. (2021)	Canada	Population-based case control study	175 EOCRC	OR for EOCRC (family history of CRC): 2.37 (1.47-3.84)	
				253 controls		
	O’Sullivan et al. (2021)		Systematic review & meta-analysis	20 studies	Pooled RR for EOCRC (family history of CRC): 4.21 (2.61-6.79)	
	Gausman et al. (2019)	USA	Retrospective, single centre cohort	269 EOCRC	Compared with controls, OR (family history of CRC): 8.61 (4.83-15.75)	
				2,802 late-onset CRC	Compared with late-onset CRC, OR: 2.87 (1.89-4.25)	
				1,122 controls		
	Kelty et al. (2019)	Australia	Retrospective population-based cohort	713,085 EOCRC,	OR: 3.6 (2.95-4.41)	
				306,329 late-onset CRC		
	Glover et al. (2019)	USA	Population-based cohort analysis (National Database- Explorys)	Definition of EOCRC: 20-39 years of age	Compared with controls, OR for EOCRC: 49.26 (42.50-57.08)	
				EOCRC rate: 18.9/100,000	Compared with late-onset CRC, OR for EOCRC: 3.6 (2.69-4.80)	
	Syed et al. (2019)	USA	Population-based cohort analysis (National Database- Explorys)	68,860 total CRC cases	Compared with late-onset CRC, OR for EOCRC: 1.78 (1.67-1.90)	
				5,710 EOCRC	Compared with controls, OR for EOCRC: 11.66 (10.97-12.39)	
**Inflammatory Bowel Disease (Ulcerative colitis and Crohn’s disease)**	Gausman et al. (2019)	USA	Retrospective, single centre cohort	269 EOCRC	EOCRC in IBD vs controls: 3% vs 0.4%, p<0.01	
				2,802 late-onset CRC	Compared with late-onset CRC, OR: 2.97 (1.16-6.63)	
				1,122 controls		
				IBD in EOCRC:		
				3 UC, 4 CD		
				IBD in late-onset CRC:		
				6 UC, 21 CD		
				IBD in controls:		
				1 UC,4 CD		
**Diabetes Mellitus**	Archambault et al. (2021)	–	Meta-analysis of 13 population-based studies.	3,767 EOCRC		No association:
				4,049 controls		OR: 1.25 (0.93-1.68)
				23,437 late-onset CRC 35,311 older controls		
	Chang et al. (2021)	Canada	Population-based case control study	175 EOCRC		No association:
				253 controls		OR: 1.75 (0.57-5.32)
	Mikaeel et al. (2021)	Australia	Prospective cohort study from The South Australian Young Onset Colorectal Polyp and Cancer Study (SAYO)	90 EOCRC (defined as patients < 55 years of age) 240 controls	OR: 4.4 (2.0-9.7)	
	Khan et al. (2020)	Germany	Population-based cohort study	101,135 diabetic patients	1.9-fold increased risk of EOCRC in diabetic patients.	
				10,698 EOCRC	6.9-fold increased risk of EOCRC in diabetic patient with family history of CRC.	
	Gausman et al. (2019)	USA	Retrospective, single centre cohort	269 EOCRC		No association:
				2,802 late-onset CRC		EOCRC vs controls: Univariate P: 0.48
				1,122 controls		
	Glover et al. (2019)	USA	Population-based cohort analysis (National Database- Explorys)	Definition of EOCRC: 20-39 years of age	Compared with controls, OR: 19.80 (18.14-21.60)	
				EOCRC rate: 18.9/100,000		

BMI, body mass index; CRC, colorectal cancer; CD, Crohn’s disease; EOCRC, early-onset colorectal cancer; IBD, inflammatory bowel disease; OR, odd ratio; RR, relative risk; UC, ulcerative colitis; USA, United State of America.

**Table 2:** Recent published studies on the dietary factors for EOCRC.

Potential Dietary Factors	Study	Country	Study Design	Participants	Comments (positive association)	Comments (no or inverse association)
**High vegetables/fruits intake**	Chang et al. (2021)	Canada	Population-based case control study	175 EOCRC	Protective effect:	No association:
				253 controls	OR (>3 vegetable servings/day): 0.52 (0.26-1.07)	OR (>3 fruit servings/day): 0.95 (0.49-1.85)
	Rosato et al. (2013)	Italy and Switzerland	Case control study	329 EOCRC 1,361 controls	Protective effect:	
					OR (high vegetable intake): 0.40 (0.28-0.56)	
					OR (high citrus fruit intake): 0.61 (0.45-0.84)	
**High fibre intake**	Chang et al. (2021)	Canada	Population-based case control study	175 EOCRC		No association:
				253 controls		OR (>3 servings/day): 1.45 (0.75-2.80)
**High red meat intake**	Archambault et al. (2021)	–	Meta-analysis of 13 population-based studies.	3,767 EOCRC	OR: 1.10 (1.04-1.16)	
				4,049 controls		
				23,437 late-onset CRC		
				35,311 older controls		
	Chang et al. (2021)	Canada	Population-based case control study	175 EOCRC		No association:
				253 controls		OR (>5 servings/week): 1.06 (0.56-1.98)
	Rosato et al. (2013)	Italy and Switzerland	Case control study	329 EOCRC 1,361 controls		No association:
						OR (high intake): 1.07 (0.79-1.47)
**High processed meat intake**	Archambault et al. (2021)	–	Meta-analysis of 13 population-based studies	3,767 EOCRC		No association:
				4,049 controls		OR: 1.03 (0.95-1.12)
				23,437 late-onset CRC		
				35,311 older controls		
	Chang et al. (2021)	Canada	Population-based case control study	175 EOCRC		No association:
				253 controls		OR (>3 servings/week): 1.23 (0.62-2.42)
	Rosato et al. (2013)	Italy and Switzerland	Case control study	329 EOCRC 1,361 controls	OR (high intake): 1.56 (1.11-2.20)	
	Archambault et al. (2021)	–	Meta-analysis of 13 population-based studies.	3,767 EOCRC		No association:
				4,049 controls		OR: 1.03 (0.95-1.12)
				23,437 late-onset CRC		
				35,311 older controls		
**High sugary drinks consumption**	Chang et al. (2021)	Canada	Population-based case control study	175 EOCRC	OR (>7 drinks/week): 2.99 (1.57-5.68)	
				253 controls		
**High Western-like diet intake**	Chang et al. (2021)	Canada	Population-based case control study	175 EOCRC	OR: 1.92 (1.01-3.66)	
				253 controls		
**Calcium supplement**	Chang et al. (2021)	Canada	Population-based case control study	175 EOCRC	Protective effect:	
				253 controls	OR: 0.53 (0.31-0.92)	
	Rosato et al. (2013)	Italy and Switzerland	Case control study	329 EOCRC 1,361 controls		No association:
						OR: 0.91 (0.64-1.29)
**Micronutrient:**	Rosato et al. (2013)	Italy and Switzerland	Case control study	329 EOCRC 1,361 controls	Protective effect:	
**Beta-carotene**					OR: 0.52 (0.37-0.72)	
**Micronutrient:**					Protective effect:	
**Vitamin C**					OR: 0.68 (0.49-0.94)	
**Micronutrient:**					Protective effect:	
**Vitamin E**					OR: 0.38 (0.26-0.58)	
**Micronutrient:**					Protective effect:	
**Folate**					OR: 0.59 (0.40-0.86)	

CRC, colorectal cancer; EOCRC, early-onset colorectal cancer; OR, odd ratio.

Chronic inflammation is another risk factor for CRC. Long-standing inflammatory bowel disease (IBD) undergoes neoplastic transformation (Low et al., 2019). Our cross-sectional study found that patients with ulcerative colitis who were diagnosed at an earlier age and suffered the disease for a long duration (>20 years) showed differentially expressed genes that are related to carcinogenesis and CRC. A large case–control study by Gausman et al. (2019), which recruited 269 patients with EOCRC, 2,802 patients with late-onset CRC, and 1,122 healthy controls, showed that a higher proportion of patients with EOCRC had IBD compared with healthy controls (3% vs 0.4%, p<0.01). Furthermore, patients with IBD nearly have a threefold higher risk of developing EOCRC than developing late-onset CRC (OR: 2.97 [1.16–6.63]).

Genetic factor is a known contributor of CRC, although only a small proportion of EOCRC has a first-degree relative with a history of CRC or adenoma (Cavestro et al., 2018). A previous study has characterized the germline mutation of cancer-susceptible genes for the hereditary subtypes of CRC (Ballester et al., 2016). **Table 3** summarizes the recent evidence (within the last 5 years of publication) of germline mutation in EOCRC. Lynch syndrome (also known as hereditary nonpolyposis colorectal cancer [CRC]) and familial adenomatous polyposis account for about 2%–4% and <1% of all CRC cases, respectively (Ballester et al., 2016; Tezcan et al., 2016). Recent studies have gained interest on the sporadic type of EOCRC; however, data are still lacking. The Cancer Genome Atlas has published the comprehensive genomic sequences of late-onset CRC (Cancer Genome Atlas, 2012). The identified mutations are located in the Wnt, MAPK, PI3K, TGF-β, and p53 pathways (Cancer Genome Atlas, 2012). A detailed genomic sequence of EOCRC is currently unavailable; therefore, linking the genotype and phenotype of the disease is difficult. A review from four cohorts involving 36,000 patients showed that the mutations of key genes in CRC biology are different between EOCRC and late-onset CRC. Catenin β1 (*CTNNB1*) and ataxia telangiectasia mutations are less likely found in EOCRC (Willauer et al., 2019). In comparison, the percentage of BRAF V600 mutation increases from ≤4% among patients with CRC aged <30 years to 13% in those aged ≥70 years (Willauer et al., 2019). A small study of 45 patients with CRC who were ≤45 years old showed normal β-catenin gene expression, which is inconsistent with the finding that 90% of sporadic CRC cases have abnormal β-catenin gene expression (Perea et al., 2010). Moreover, the majority of tumor samples (70%) lack cyclin E expression, which might be associated with poor prognosis (Perea et al., 2010). Thus, validation in a bigger number of samples is required to confirm all the genomic changes in EOCRC.

**Table 3:** Recent published studies on germline mutations in EOCRC patients.

Published studies	Country	Study Design	Participants	Comments
Lieu et al. (2019)	USA	Next-generation sequencing	4,668 EOCRC	Microsatellite stable cohort:
			13,550 late-onset CRC	a) TP53 and CTNNB1 were more common in EOCRC
				b) APC, KRAS, BRAF and FAM123B were more common in late-onset CRC
				Microsatellite instability high cohort:
				a) APC, BRAF and KRAS were more common in EOCRC
Willauer et al. (2019)	USA	Next-generation sequencing	1,162 EOCRC	As compared to late-onset CRC, EOCRC more likely to have:
			2,583 late-onset CRC	a) microsatellite instability (P=0.038) b) fewer BRAF V600 mutations (p<0.0001) As compared to late-onset CRC, EOCRC had higher frequency of CMS1 (22-23% vs 11%) and lower frequencies of CMS2 (43% vs 50%) and CMS4 (20-22% vs 27%).
Stoffel and Murphy (2019)	USA	Next-generation sequencing	79/315 EOCRC had gene mutations associated with hereditary cancer syndrome	a) 56 Lynch syndrome (MSH2, MLH1, MSH6, PMS2) b) 10 familial adenomatous polyposis (APC, MUTYH) c) 13 mutations in other cancer-associated genes (MUTYH, SMAD4, BRCA1, TP53, CHEK2)
			21/315 EOCRC had variants of uncertain significance	Only 51% of the subjects with germline mutations associated with hereditary cancer syndrome reported a family history of CRC.
Pearlman et al. (2017)	USA	Next-generation sequencing	72/450 EOCRC had gene mutations	48 (10.7%) had MMR-deficient tumors and 40 (83.3%) had at least 1 gene mutation:
				a) 37 Lynch syndrome (MLH1, MLH2, MSH6, PMS2) b) 1 APC c.3920T>A, p.l1307K & PMS2 c) 9 double somatic MMR mutations d) 1 somatic MLH1 methylation 402 (89.3%) had MMR-proficient tumors and 32 (8%) had at least 1 gene mutation: a) 9 high-penetrance CRC genes (APC, APC/PMS2, MUTYH, SMAD4) b) 13 high-penetrance other cancer-associated genes (ATM, BRCA1, BRCA2, CDKN2A, PALB2) c) 10 low-penetrance CRC genes (APC c.3920T>A, p.l1307K, monoallelic MUTYH)
Perea et al. (2017)	Spain	Quantitative real-time PCR	60 EOCRC	a) 16p13.12-p13.11 alterations were more prevalent in EOCRC (33.3% vs 16.3%). b) 100% (34/34) EOCRC showed homozygous deletion in NOMO-1 gene, as compared to late-onset CRC, 2/17 (11.7%). c) microsatellite stable EOCRC showed high proportion of homozygous deletion in NOMO-1 gene (91.5%).
			86 late-onset CRC	
Arriba et al. (2016)	Spain	Array comparative genomic hybridization profiling	60 EOCRC	Chromosomal instability profiles:
			86 late-onset CRC	a) EOCRC: losses at 1p36, 1p12, 1q21, 9p13, 14q11, 16p13, 16p12 b) late-onset CRC: gains at 7q11, 7q22
Watson et al. (2016)	USA	Next-generation sequencing, immunohistochemistry and PCR	68 EOCRC (patients ≤40 years of age)	Higher proportion of EOCRC (54%) harbored KRAS mutation, independent of tumor stage.

APC, adenomatous polyposis coli; ATM, A-T mutated; CDKN, cyclin dependent kinase inhibitor; CMS, congenital myasthenic syndromes; CRC, colorectal cancer; CTNNB, catenin beta; EOCRC, early-onset colorectal cancer; KRAS, Kirsten rat sarcoma; MLH, mutL homolog; MMR, mismatch repair; MSH, mutS homolog; MUTYH, mutY DNA glycosylase; PALB2, partner and localizer of BRCA2; PMS, postmeiotic segregration increased; SMAD, mothers against decapentaplegic; TP, tumor protein; USA, United State of America.

The classical theory of the pathogenesis of CRC was reported to be similar to that of late-onset CRC. The adenoma–carcinoma sequence explains the accumulation of mutations from normal colonic or rectal mucosa to the precursor of an adenomatous polyp and eventually carcinoma formation. A 7-year follow-up of 119 EOCRC cases demonstrated that 53% developed into polyps, mostly in the right colon with good prognosis because of early diagnosed; the remaining 47% had no polyps and were diagnosed at an advanced stage (Perea Garcia et al., 2019). The authors concluded that molecular markers, as well as surveillance for a longer period of time, are needed to detect polyp development.

No definite risk factor can be solely attributed to the pathogenesis of EOCRC; thus, the pathogenesis is more likely due to a complex interaction of the multiple elements involved. Early-life exposure, such as the mode of delivery, mode of nutritional intake (breastfeeding vs diet formula), antibiotics use, and maternal well-being, might contribute to the early carcinogenesis of EOCRC (Hofseth et al., 2020). Subsequently, childhood, adolescence, and early adulthood exposures, namely, lifestyle habits, such as diet, exercise, smoking, and alcohol consumption, together with chronic health conditions, such as diabetes mellitus, IBD, and obesity, will come into play. All these environmental exposures will cross-interact within genetically susceptible individuals and lead to EOCRC. Another important player in this complex interaction is gut microbiota. The composition of microbiota can be influenced by various environmental exposure and, in turn, contributes to the development of EOCRC (Akimoto et al., 2020).

Advancement in high-throughput microbiome sequencing and mass spectroscopy has enabled researchers to characterize individualized oncogenic microbiomes and their metabolites (Bechshoft et al., 2016; Wirbel et al., 2019; Yachida et al., 2019), which colonize at tumor and non-tumor colonic sites. However, the variation in the use of the amplicon sequencing platforms and analysis procedures during experimental design related to gut microbiota and CRC differ from one study to another, which makes the analysis of gut microbiota in CRC even more challenging (Mo et al., 2020). Additionally, the types of specimens used, and the timing of sample collection raise the difficulty of attaining a final list of gut microbiota specifically for CRC (Mira-Pascual et al., 2015; Thomas et al., 2016). For over a decade, studies have reported on the relationship between gut dysbiosis and CRC (Nakatsu et al., 2015; Fan et al., 2020). Gut dysbiosis is characterized by an alteration of bacterial species that leads to the imbalance between beneficial and pathogenic bacteria (Nakatsu et al., 2015; Fan et al., 2020). Inevitably, the scientific premise that supports the mechanistic link between gut microbial dysbiosis and CRC is strong; specific microorganisms have been identified exert a key role in colorectal carcinogenesis through various mechanisms, such as intestinal dysbiosis, inflammation, evasion of tumoral immune response, and the activation of pro-tumoral signaling pathways, such as β-catenin (Hernandez-Luna et al., 2019).

## Gut Microbiota Dysbiosis in EOCRC

The gut microbiota is intimately involved in numerous aspects of normal host physiology, from nutritional status to behavior and stress response. It is directly involved in the maintenance of mucosal homeostasis, epithelial barrier function (Sekirov et al., 2010; Hofseth et al., 2020), and protection against pathogenic challenge (Marchesi and Shanahan, 2007). Under normal condition, the intestinal barrier efficiently compartmentalizes bacteria to the lumen with minimal penetration to the mucosa (Marchesi and Shanahan, 2007), but perturbations in gut barrier function can lead to increased intestinal permeability (Saus et al., 2019).

The imbalance or disturbance patterns of the gut microbiota have been recognized as an indicator of a given disease or poor health status (Shreiner et al., 2015). Theoretically, the enrichment of several bacterial species in the gut contributes to colorectal carcinogenesis by inducing tumor proliferation, promoting inflammation, causing DNA damage, and protecting tumor from immune attack. By contrast, some bacteria, mostly probiotics, are depleted in patients with CRC (Yu, 2018; Fong et al., 2020). Another theory suggested that altered luminal microbiota may occur following inflammation. Gut microbiota composition in mice model of colitis induced by genetically deleting interleukin-10 signaling revealed high levels of luminal Verrucomicrobia, Bacteriodetes, and Proteobacteria at the phylum-level as compared to control (Arthur et al., 2012). This is the possible explanation of how inflammation could generate an environment that favors carcinogenesis by altering the composition of gut microbiota. At the moment, there is no gold standard exists for the determination of dysbiosis or the extent of gut microbiota imbalance because of the huge inter-individual variation among the healthy population (Wei et al., 2021). Moreover, the source of pathobionts and the emergence of disease-associated microbiota either related to diet, genetic, immune or barrier-related factors were still unclear (Olesen and Alm, 2016). A variety of different dysbiosis indexes have been suggested and applied to characterize diseases and adverse conditions, predict treatment outcomes, and provide information other than the commonly used alpha and beta diversity assessments (Wei et al., 2021).

The earliest data concerning the gut microbiota were generated using cultural approaches that lack information related to ecosystem evaluation because less than 30% of intestinal bacteria have been cultivated (Lagier et al., 2016). The advent of molecular tools that target the bacterial 16S ribosomal RNA (rRNA) gene has revolutionized the knowledge of gut microbiota from feces and tissues without the need for cultivation approach. Genetic fingerprinting techniques, such as terminal restriction fragment length polymorphism; denaturing gradient gel electrophoresis; and hybridization approaches, such as fluorescence *in situ* hybridization, microarrays, and clone library analysis, have been applied to provide a more complete description of the gut microbiota’s genomic structures (Villeger et al., 2018). Quantitative polymerase chain reaction and 16S rRNA gene next-generation sequencing are the current methods used for describing the composition of the intestinal bacterial community and comparing the gut microbiota of patients with EOCRC and CRC from that of healthy individuals. In addition, long-read sequences generated using MinION sequencing can compensate for the low numbers of reads for bacterial classification (Taylor et al., 2020).

A detailed enumeration of bacterial composition according to host phenotypes will help shed light into the relationship between gut microbiota and CRC carcinogenesis. A recent study utilized machine learning-based method to investigate the microbial differences among CRC, colorectal adenoma, and healthy control groups using the 16S rRNA data sets retrieved from 15 studies (Mo et al., 2020). This study reported that the dysbiosis patterns of adjacent tissues of late-onset CRC and colorectal adenoma are similar with fluctuations in the relative abundances of genera *Pseudomonas, Streptococcus, Porphyromonas*, and *Fusobacterium*, compared to healthy control. The microbiome dysbiosis pattern of the adjacent tissue and adenomas were highly correlated with dysbiosis pattern in CRC tissue (Mo et al., 2020). Although this study suggested the putative utility and validity of microbiota-based CRC risk assessment as diagnostic markers in discriminating healthy and diseased individuals, data that describe GM during EOCRC are still scarce.

Nevertheless, evidence has proven that EOCRC occurs mostly at the rectal area (Archambault et al., 2021), and if found in the colon, EORCR usually occurs in the distal colon (Patel and Ahnen, 2018). Aitchison et al. (2020) reported that EOCRC tumors are linked to high adenomatous polyposis coli (*APC*) gene mutations and located mostly at distal areas, although *Fusobacterium nucleatum* is often found to be more abundant in proximal colon tumors compared with distal colon and rectal tumors (Liu L. et al., 2018). In a case–control study, (Bullman et al., 2017) showed that *F. nucleatum* is abundant in paired primary colorectal tumors and corresponding liver metastases in the cecum and ascending and in unpaired colorectal tumors and liver metastases in the rectal area. Previously, a method using random forest prediction was proposed based on the abundance of specific operational taxonomic unit and area under the curve (AUC) value. This method has been used to analyze symptoms associated with functional gastrointestinal disorders (Saffouri et al., 2019) and to detect a fecal biomarker for CRC in two Asian cohorts (Guo et al., 2018). Specifically, this approach managed to distinguish between patients with CRC and healthy controls by highlighting the remarkable increase in the relative abundance of *F. nucleatum*. Remarkably, this study also observed the trajectory of the early disease stage by investigating five AUCs to distinguish EOCRC patients (stage, I + II) from healthy controls (Kosumi et al., 2018).

*F. nucleatum, Bacteroides fragilis* and *Escherichia coli* are the frequently reported gut bacteria related to late-onset CRC. *F. nucleatum* is an obligate, anaerobic oral commensal bacterium normally found in human oral cavity, upper respiratory tract, and intestinal tract (Li et al., 2016; Amitay et al., 2017). It is remarkably elevated in patients with CRC and lymph node metastasis and associated with poor survival (Li et al., 2016; Amitay et al., 2017; Bullman et al., 2017; Matsumoto et al., 2019). The carcinogenic mechanisms induced by microorganism is basically strain specific. For example, *F. nucleatum* is a tumor microbiota that contributes to the cancerous microenvironment in the gut (Bullman et al., 2017; Zhou et al., 2018). *F. nucleatum* can easily bind to the host and cause DNA damage because of its several virulence factors, such as FadA, Fap2, and MORN2 proteins (Jackson and Bartek, 2009; Ranjbar et al., 2021). In an animal model, the upregulation of *chk2* promoted DNA damage and the progression of *F. nucleatum*-induced CRC (Guo et al., 2020). On the other hand, *Escherichia coli* (*E. coli*), a Gram-negative, facultative, anaerobic bacteria, normally found in healthy human gut (Yu, 2018). Cevallos et al. (2019) and Genua et al. (2021) postulated high association of *E. coli* and CRC by taking opportunity of intestinal barrier damage, given the right condition and time. *E. coli* harbor polyketide synthases (pks) pathogenicity island which encodes for genotoxins (Colibactin) that can damage DNA and enhances intestinal epithelial permeability (Arthur et al., 2012; Tomkovich et al., 2017; Yu, 2018). The growth of colibactin *E. coli* is greatly enhanced with increased epithelial oxygenation, which is highly associated with tumorigenesis (Cevallos et al., 2019). *E. coli* may also develop tumorigenicity in a healthy human gut over time and may contribute to CRC by adjusting host’s epithelial autophagy and oxidative stress response (Yu et al., 2021). However, invasiveness of E. coli can be countered by PTPN2 gene which encodes for T-cell protein tyrosine phosphatase (TCPTP) (Shawki et al., 2020). Increased inflammatory activity through Janus kinases and signal transducer and activator of transcription (JAK-STAT) pathway, and significant barrier defects were noted in *PTPN2* knockout mice. Interestingly, Nakkarach et al. (2021) highlighted anti-inflammatory and anti-cancer effects of *E. coli* through the production of short chain fatty acids, and reduction of nitric oxide and proinflammatory cytokines.

In a Western cohort study, the incidence of late-onset CRC is higher in African–Americans compared to the non-Hispanic whites because of their dietary intake of high animal protein and fat that are enriched with sulfidogenic bacteria (Yazici et al., 2017). Intestinal *Bilophila wadsworthia*-specific *dsrA* is anticipated to promote CRC among African–American population, because it is associated with hydrogen sulfide, which triggers pro-inflammatory pathways and hyperproliferation (Dahmus et al., 2018). *B. fragilis* is an obligate, anaerobic commensal bacterium normally found in the colon. *B. fragilis* has two types, namely, non-toxigenic (NTBF) and enterotoxigenic *B. fragilis* (ETBF). ETBF produces *B. fragilis* toxin (BFT), which is associated with inflammation and CRC development (Boleij et al., 2015). BFT has three isotypes coded by the *bft* gene (bft-1, bft-2, and bft-3), among which bft-2 is the most common and most pathological isotype found in patients with CRC because of its enhanced mucosal adherence ability (Boleij et al., 2015; Jasemi et al., 2020). The *bft* gene isotypes are located at the pathogenicity island region of ETBF strains. The effectiveness of *B. fragilis* in inflammation and colon carcinogenesis development depends highly on its biofilm-forming ability, as well as the availability of the flanking region and pathogenicity island region of certain strains. Biofilm formation creates a barrier that prevents antibiotic access and aids in adherence to epithelial surface, which increase the chances of the survival of *B. fragilis*. However, biofilm-forming ability is not related to the availability of the *bft* gene. EFBF strains are more potent than NTBF because of these characteristics (Jasemi et al., 2020). BFT binds to a specific receptor on intestinal epithelial cells (IEC, ATP dependent), which leads to the activation of the Wnt and NF-kβ signaling pathways and eventually leads to the increase in cell proliferation, DNA damage, and the release of inflammatory mediators, especially IL-17 (Boleij et al., 2015; Jasemi et al., 2020). *B. fragilis* prefers to colonize epithelial crypts, which creates a safer colonization because it can evade the host’s immune attack (Boleij et al., 2015).

Speculations have been made regarding the relation of *Akkermansia muciniphila* with CRC. The abundance of *A. muciniphila* is negatively correlated with overweight and obesity (Matsumoto et al., 2019). *A. muciniphila* is thought to trigger the onset of CRC through the degradation of mucosal layer, which then creates an access to IEC and induces hyperproliferation (Ijssennagger et al., 2015), because of its relation with obesity (Liu et al., 2019; Siegel et al., 2019). However, we are not yet certain that *A. mucinophila* is the cause of CRC. A randomized, double-blind clinical trial of 32 volunteers demonstrated the beneficial effects of *A. muciniphila*, such as the improvement of insulin sensitivity and the reduction of insulinemia and plasma total cholesterol. This study also showed unremarkable reduction in hip circumference, fat mass, and body weight in the volunteers with *A. muciniphila* supplementation (Depommier et al., 2019). Other bacteria, such as *Prevotella* and *Bacteroides*, are commonly found in the gut and may be involved in the onset of cancer. Although *Prevotella* is related to high-fiber diet, it is also associated with diet high in red meat (Ijssennagger et al., 2015). However, its association to cancer has not been elucidated.

Despite gut microbiota provides evidence for the exposome in developing CRC, the overall picture of the roles of GM and host’s interaction coincide during EOCRC is still unclear (Hofseth et al., 2020). First, it is impossible to pinpoint the exact microenvironment, host’s immune response and pathogens or pathobionts involved, in the initiation of EOCRC due to the complexities of human-microbe interplay. Studies using fecal or tissue samples at a single time point, and experiments *in vivo* and *in vitro* may not accurately constitute the actual occurrence in EOCRC. Secondly, it must be noted that there are other confounding factors such as genetic, environmental factors, diet, lifestyle, the use of antibiotics, and immunotherapy may play a part in the EOCRC process (Yu, 2018). Furthermore, viruses and fungi are also a part of the intestinal microbiota; however, their role in EOCRC and CRC is not fully understood due to their intricate host cell invasion and immune evasion pathways. (Collins et al., 2011). Therefore, the key species involved in EOCRC progression are thought to differ based on exposomal elements, such as stress, dietary habits, ethnicity, and geographical differences (Senghor et al., 2018; Zhao et al., 2019).

### Gut Microbiota Metabolites in EOCRC

Accumulating studies proposed that specific pathogens among the intestinal microbiota have procarcinogenic activities (Kostic et al., 2013; Wu et al., 2019; Haruki et al., 2020). Metabolic products derived from microbial community may also contribute to the etiology of CRC (Louis et al., 2014; Hashim et al., 2021). Specific microbial community or pathogen plays a key role in initiating or exacerbating tumorigenesis by inducing chronic inflammation, suppressing immunosurveillance, and producing oncogenic metabolites (Wong and Yu, 2019). Mass spectrometry and nuclear magnetic resonance spectrometry are the two main methods applied in metabolomics. Mass spectrometry is becoming more widely used in host–microbiota research because of its high sensitivity, high throughput, and applicability to a greater variety of metabolites. Therefore, comprehensive metagenomic and metabolomic analyses might provide an alternative approach to understand CRC development through associated changes in the gut environment caused by dysbiosis. Some of the functions of short chain fatty acids (SCFAs) are negatively correlated with inflammation and cancer (Nakkarach et al., 2021). Other microbial metabolites, such as bile acids and its derivatives, are also associated with carcinogenesis (Ajouz et al., 2014).

Gut microbiota in healthy humans plays an important role in gut homeostasis and has an impact on host metabolism. SCFA is one of the post-biotic component that contributes greatly to maintain gastrointestinal tissue integrity and may positively affect the body’s immune response (Patil et al., 2019). In the colon, SCFAs, such as butyrate, acetate, propionate, and valerate, are produced from fiber fermentation (Vital et al., 2014). These SCFAs represent a major energy source for colonocytes and act as regulators of immune response (Tilg et al., 2018). Additionally, SCFAs play a positive role in intestinal barrier function by promoting mucus production and connexin expression. It also supports the health of gut microbiota by increasing microbial diversity (Fan et al., 2020). Dietary fiber deprivation induces the growth of mucus-eroding bacteria leading to mucosal barrier dysfunction (Desai et al., 2016). Moreover, several bacteria have been identified as potential butyrate producers (Vital et al., 2014). Butyrate is linked with the high prevalence of CRC among African/Caucasian American population (O’Keefe, 2016). It has a protective role in CRC, where the depletion of butyrate-producing bacteria species and diminished fecal butyrate levels are associated with colon tumorigenesis (Peng et al., 2021). Butyrate is among the most important SCFA derivatives that function in anti-inflammation and antitumor through cell metabolism, microbiota homeostasis, immune regulation, and epigenetic modulation (Makki et al., 2018; Fan et al., 2020). Chen et al. (2013) and Ou et al. (2013) presented that an increased abundance of *Enterococcus*, *Streptococcus* spp., and *Bacteroides* enterotypes are negatively correlated with the reduction of butyrate among the studied populations with CRC. The correlation between butyrate reduction and EOCRC has not yet been established. However, the authors anticipated that the accruing evidence of butyrate reduction patterns among EOCRC warrant further investigation.

Liver produces primary bile acids, such as cholic acid and chenodeoxycholic acid from cholesterol in hepatocytes, and subsequently produces secondary bile acids, such as deoxycholic acid and lithocholic acid, which are strongly correlated to CRC (Flint et al., 2012; Yachida et al., 2019). The accumulation of bile acids is postulated to be the result of a high consumption of fat and animal protein and a low intake of fiber in a daily diet (Breuer and Goebell, 1985). Most protein fermentation products, such as hydrogen sulfide and nitroso compounds, are toxic to intestinal cells and are therefore implicated in the etiology of CRC (Jensen et al., 2016; Diether and Willing, 2019). Sulfur-metabolizing microbes, such as *B. wadsworthia*, *Streptococcus bovis, Helicobacter pylori, B. fragilis*, and *Clostridium septicum*, have a tendency to convert the dietary sources of sulfur into genotoxic hydrogen sulfide (Dahmus et al., 2018; Tilg et al., 2018), which is associated with the development of CRC (Nguyen et al., 2020). In addition, low-carbohydrate–high-protein diets appear to have detrimental effects on gut microbiota, with potentially negative long-term health consequences for the host (Korpela, 2018). Chronic exposure to high bile acid levels is associated with the generation of reactive oxygen and nitrogen species, the disruption of the cell membrane and mitochondria, the induction of DNA damage and mutations, and the development of reduced apoptosis capability (Fan et al., 2020).

The main bacterial genera involved in secondary bile acid biosynthesis are *Bacteroides*, *Clostridium*, *Lactobacillus*, *Bifidobacterium*, and *Eubacterium* (Peng et al., 2021). These bacteria also serve key roles in regulating host fat metabolism (Peng et al., 2021). Moreover, a study reported that *B. wadsworthia* abundance is positively correlated with deoxycholic acid accumulation in patients with multiple polypoid adenomas and intramucosal carcinoma (Yachida et al., 2019). The administration of deoxycholic acid induces colonic tumor formation and is considered a substantial contributors to the development of CRC, particularly in the context of obesity (Bernstein et al., 2011). Metabolites, such as butyrate and indole-3-propionic acid, may confer positive effects on gut health by improving the intestinal barrier (Khan et al., 2014; Xiao et al., 2020; Li et al., 2021). Other metabolites, including deoxycholic acid, spermine, and trimethylamine N-oxide, increase the risk of cancer (Peng et al., 2021; Tan et al., 2021). The mechanism by which bile acids affect the progression of EOCRC is unknown. These findings highlight the pressing need to understand the complex molecular mechanisms and validate the causality of these metabolites among EOCRC.

## Factors Influencing the Pattern of GM Dysbiosis in EOCRC

Alterations in microbiota can result from diet, toxins, drugs, and pathogens. Among these factors, enteric pathogens have the greatest potential to cause microbial dysbiosis (Carding et al., 2015). Evidences suggested that the microbial shift due to dysbiosis shapes the host’s physiological functions and therefore leads to the pathogenesis of intestinal and extra-intestinal disorders (Ley et al., 2005; De Palma et al., 2010; Ahmad et al., 2020).

### Diet Related to Dysbiosis in EOCRC

Diet containing high amounts of fat and sugar (known as inflammatory diet) may damage the intestinal microenvironment, which leads to inflammation and may subsequently destructs the intestinal epithelial barrier. The exact mechanism underpinning this process is presently unknown but is posited to the metabolic decomposition of lipids, such as secondary bile acids and hydrogen sulfide. Zhao et al. (2020) demonstrated that inflammation in mice with induced colitis can be reduced by farnesoid X receptor, a nuclear receptor regulated by bile acids. Liu L. et al. (2018) previously established that the exposure of IECs to bacterial components triggers the formation of hyperplasia and polyp growth. Hamilton et al. (2015) compared rats fed with high-fat diet with those on normal diet and revealed that an increase in *Bacteroides* in the rats fed with high-fat diet compromised intestinal integrity. Regular red meat consumption increases the risk of CRC as red meat contains heme, which induces toxicity and damages the intestinal epithelium (Hamilton et al., 2015; Pignatelli et al., 2021). Epithelial damage leads to compensatory hyperproliferation and eventually causes hyperplasia (Ijssennagger et al., 2015). Processed meat (preserved meat by curing, salting, smoking, and canning) also induces inflammation, because it contains high amounts of saturated fats, trans fats, and cholesterol. The curing process results in the release of endogenous N-nitroso compounds (NOCs), such as nitrates and nitrites (Mehta et al., 2019; Pignatelli et al., 2021). Mehta et al. (2019) found that the overall risk of developing EOCRC also depends on the cooking method of the consumed meat. In particular, cooking meat at high temperature will release polycyclic aromatic hydrocarbons (PAHs) and heterocyclic amines (HCAs), which, together with NOCs, trigger mutations in pro-inflammatory genes, stimulate DNA damage through alkylation, and lead to tumorigenesis (Mehta et al., 2019).

Young, obese adults are at higher risk of developing CRC (Liu et al., 2019). For example, (Low et al., 2020) demonstrated that the symptoms of patients with EOCRC improves upon weight loss, which suggests that obesity, sedentary lifestyle, and dysbiosis are interrelated. Nonetheless, whether obesity is a risk or a causal factor of dysbiosis has no consensus (Mehta et al., 2019; Siegel et al., 2019). Notably, the association between obesity and CRC is stronger for individuals below 50 years of age, which suggests the higher risk for developing EOCRC among young obese adults (Liu et al., 2019).

### Mental Health Status and Dysbiosis in EOCRC

Stress as a key factor that contributes to EOCRC affects eating pattern and lifestyle, which in turn shape the gut microbiota and cancer development (Zhang et al., 2018). The epigenetic make-up, body immune system, and gut microbiota diversity of offspring are influenced by maternal stress and associated sleeping disorders. A 5-year cohort study on Japan’s population revealed an 11% increase in cancer risk for individuals with a constantly high perceived stress level in the long term (Song et al., 2017). However, these results are more confined to men because of the interplay of other risk factors, such as obesity, smoking, and alcohol consumption. Blum-Barnett et al. (2018) reported that EOCRC survivors suffer physically and emotionally, which result in reduced quality of life and increased risk of developing depression. Physical side effects comprise those from cancer itself and the treatment, whereas emotional side effect includes relationship challenges and the lack of resources to assist in coping with the aftermath of CRC. The authors also concluded that individuals aged 50 years and less commonly face financial burden, which increases their risk of developing EOCRC compared with individuals of more than 50 years old.

## Cross-Talk Between GM and Host Phenotype

The key to a good relationship between the host and gut microbiota is a healthy intestinal barrier. Some gut flora and humans have a mutualistic interaction rather than just a commensal interaction. Some microbes in the human gut help the host by digesting dietary fiber into SCFAs, such as acetate, propionate, and butyrate, for easier absorption by the host (Nakkarach et al., 2021).

### The Physical Barrier in Gut

Gut microbiota and human intestine are separated by a physical barrier. The physical barrier is made up of a layer of IECs and covered by a mucus layer. A layer of connective tissue, called the lamina propria, lies beneath the intestinal epithelium layer (Parveen et al., 2021). IECs comprise highly specialized cells, such as enterocytes, endocytes, goblet cells, and Paneth cells, which are arranged tightly in a single layer by tight junctions, adherence junctions, and desmosomes. Goblet cells secrete oligomeric, mucus gel-forming proteins known as mucin. A network of claudins and occludins, which intermingle with zonula occludens proteins, forms the tight junction complex (Parveen et al., 2021). Claudin-1 overexpression is associated with an increase in cell proliferation and the expression of inflammatory genes, which result in increased tumor growth and size, reduced mucosal permeability, and poor survival (Pope et al., 2014). Together with the IECs and mucosal layer, these structures protect the human gut from direct exposure to microbes and selectively permit the absorption of water, electrolytes, and nutrients (Parveen et al., 2021).

The mucin family is consisted of 22 mucins (Lu et al., 2019; Gan et al., 2020). Mucin may be membrane-bound mucin or secretory mucin, which is further divided into gel-forming and soluble types. Secretory mucins are consisted of mucin 2 (MUC2), MUC5, MUC6, MUC7, MUC8, MUC9, and MUC19, which are generated by goblet cells and extracted out into the lumen of the intestine to form a physical barrier. Membrane-bound mucins are consisted of MUC1, MUC3, MUC4, MUC12, MUC13, MUC15, MUC16, MUC17, MUC18, MUC20, MUC21, and MUC22, which are anchored to the cell surface of the intestinal epithelium. MUC4, MUC17, MUC20, and MUC21 are transmembrane mucins. These mucins protect the human gut from microbes and inflammation by forming protective mucous gel through O-glycosylated tandem repeats that extend into the mucous gel (Lu et al., 2019; Gan et al., 2020). Transmembrane mucins are also important for cell-to-cell and cell-to-microenvironment communication. They are associated with the overall survival and event-free survival of patients with non-metastatic CRC (Lu et al., 2019).

The overexpression of MUC1, a heterodimeric protein, activates inflammatory pathways through NF-kβ p65 and MYC signaling, which leadings to tumorigenesis and further increases MUC1 expression (Li W. et al., 2020). MUC1 and MUC3 are glycoproteins that contain O-linked oligosaccharides (i.e., O-glycan) (Bergstrom et al., 2016). A study on a mice model showed that the lack of these intestinal cores leads to severe inflammation and an earlier onset of colon tumors. MUC 2, a high-molecular-weight glycoprotein encoded and produced by *MUC2* gene, is a major contributor to the mucosal barrier (Kumar et al., 2017). The loss of *MUC2* causes inflammation in the gut and increases intestinal barrier permeability (Gan et al., 2020; Parveen et al., 2021). MUC4, a large transmembrane mucin, is naturally expressed in the small and large intestines and differentially expressed in colitis and colitis-associated cancer (Das et al., 2016). MUC4 is also linked to tumorigenesis in the colon by stimulating proinflammatory cytokines. In this experimental study, mice with increased *MUC4* expression have poorer survival (Das et al., 2016). Nevertheless, mice that lack *MUC4* expression, which is equivalent to MUC17 in humans, express more *MUC2* and *MUC3*. *MUC4* is essential to maintain the integrity of the intestinal barrier (Das et al., 2016). Mice that lacking *MUC4* seems to form a resistance against the development of colitis and colitis-associated diseases (Das et al., 2016). Study using mice model implicated the involvement of MUC5AC, with help from CD44, in colorectal carcinogenesis (Pothuraju et al., 2020). CD44 is a transmembrane glycoprotein that regulates cell migration, invasion, and metastasis. MUC6 has been associated with good prognosis in patients with CRC (Lu et al., 2019). An increased expression of MUC20 is related to high recurrency and poor survival in patients with CRC (Lu et al., 2019).

## Gut Dysbiosis and Intestinal Barrier Dysfunction

### Diversity of GM in EOCRC

The role of GM in maintaining physical barrier function and preventing disease progression is undeniably crucial. The imbalance of beneficial and detrimental bacteria causes dysbiosis. A reduction in microbiota diversity allows opportunistic pathobionts to infiltrate the gut *via* both transcellular and paracellular pathways (Yu, 2018). This resulting in weakening of the physical barrier of the gut. In acute-phase reaction, TNF-α plays the main role in exacerbating inflammation and increasing epithelial cell permeability. TNF-α binds to TNF receptor 2 expressed on IECs and induces the myosin light chain kinase (MLCK) signaling pathway, which will eventually increase epithelial permeability (Muzzi et al., 2021). TNF-like 1A, a novel TNF superfamily member, was reported to be up-regulated in colonic biopsy of IBD samples (Pai et al., 2021). The up-regulation of the gene triggered intraepithelial passage of bacteria *via* PI3K/Akt/MLCK2 pathway prior to the tight junction damage (Pai et al., 2021). Glucocorticoid receptors, also available on IECs, can prevent this occurrence (Muzzi et al., 2021). Certain chemokines, such as CXCL1, will not be stimulated without glucocorticoid receptors; thus, more microbes can infiltrate and aggravate the disease course. The absence of glucocorticoid receptors showed increased inflammation by the induction of toll-like receptor 4, which leads to the increased activation of NF-kβ signaling by the invading bacteria. Li et al. (2019) tested the role of GM. Mice fed with the feces of patients with CRC contain remarkably higher pathogenic bacteria and lesser SCFA-producing bacteria, which led to a reduction in SCFA production. Furthermore, these mice have increased tumor proliferation and growth and reduced tumor cell apoptosis compared with mice fed with the feces of healthy patients. Moreover, the mice have distraught intestinal barrier function, increased pro-inflammatory cytokine expression, and increased β-catenin and cyclin D1 expression, which lead to the activation of Wnt signaling and eventually tumorigenesis.

### Molecular Pathway Related to EOCRC

*F. nucleatum* activates CTNNB1 signaling through the binding of its virulence factor, FadA, to CDH1 (E-cadherin) (Li et al., 2016; Amitay et al., 2017; Bullman et al., 2017; Liu L. et al., 2018). Activated β-catenin (Wnt signaling pathway) leads to the increased release of transcription factors, oncogenes, and inflammatory cytokines (TNF-α and IL-10). *F. nucleatum* inhibits T-cell-mediated immune responses, increases genetic mutation (BRAF, KRAS, TP53, CHD7, and CHD8), induces CpG island methylator phenotype, and increases microsatellite instability (MSI), which will eventually lead to tumorigenesis (Li et al., 2016; Amitay et al., 2017). The oncogenicity of *F. nucleatum* is due to its ability to adhere and invade epithelial cells and release RNA into host’s cells (Li et al., 2016). *F. nucleatum* is highly associated with increased risk of developing advanced CRC stage by promoting inflammation, especially when supported by inflammatory diets (high fat and sugar) (Bullman et al., 2017; Liu L. et al., 2018). In another case–control study by Amitay et al. (2017), *F. nucleatum* is abundant in the feces of participants with CRC but not in participants with adenomas or advanced adenomas. The availability of *F. nucleatum* may be used as a monitoring strategy in the treatment and management of CRC.

Inflammatory diets, mutagens (NOCs, PAHs, and HCAs), and heme from red meat can damage the intestinal barrier by promoting sulfide-producing bacteria and mucin-degrading bacteria, such as *A. muciniphila* (Ijssennagger et al., 2015; Mehta et al., 2019; Pignatelli et al., 2021). Ijssennagger et al. (2015) established that the mucosal layer of denatured mucins may become thinner because of the breakage of disulfide bond by microbial hydrogen sulfide, which exposes the epithelial cells to microbes (Ijssennagger et al., 2015). This cascade of events results in the activation of the Wnt signaling pathway, which increases the risk of CRC. Liu L. et al. (2018) and Pignatelli et al. (2021) found that *F. nucleatum* triggers colorectal tumorigenesis through the activation of the Wnt pathway (Liu L. et al., 2018). The abundance of *F. nucleatum* at tumor area results in the increased production of reactive oxygen species, which initiate mutation through the reduction of MLH1 expression and subsequent MSI (Pignatelli et al., 2021). In addition, *F. nucleatum* increases metalloproteases, which activate inflammatory pathways (Pignatelli et al., 2021). According to Liu L. et al. (2018), APC and RNF43 play a role in the stabilization and nuclear translocation of β-catenin in the Wnt pathway. Similarly, Liu L. et al. (2018) reported that APC mutation may induce adenocarcinoma and mucous adenocarcinoma. Conversely, according to Li et al. (2020a), signet ring cell carcinoma is related to RNF43 mutations. An ample body of evidence indicate that increased Wnt signaling may lead to p53 and p21 reduction, which results in diminished cell cycle arrest, lessened apoptosis, and finally the advancement of cancer (Li et al., 2020a). Although the Wnt pathway seems to be the key to tumorigenesis, the high proportion of APC mutations found outside the mutation cluster region suggests that β-catenin activation is not remarkable in EOCRC (Aitchison et al., 2020).

**Figure 1:**
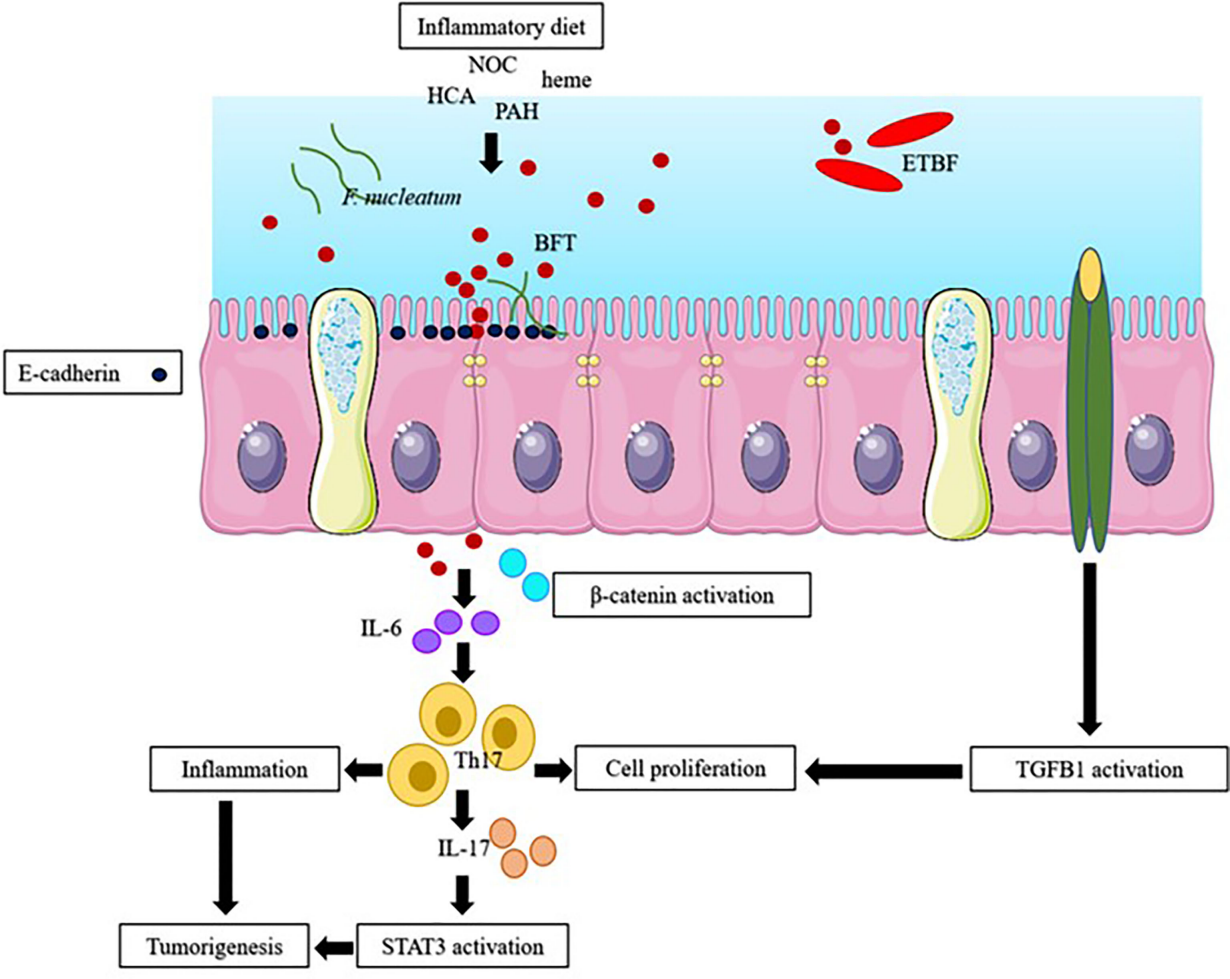
The possible pathway of early-onset colorectal cancer (EOCRC). Inflammatory diet (high fat and sugar diet), endogenous N-nitroso compound (NOC), polycyclic aromatic hydrocarbons (PAH), heterocyclic amines (HCA), heme, stress, *F. nucleatum* and *B. fragilis* toxin (BFT), produced by enterotoxigenic *B. fragilis* (ETBF), induced inflammation through stimulation of inflammatory cytokines and causes (i) the activation of β-catenin and STAT3 signaling pathway; and the activation of transforming growth factor beta 1 (TGFB1) pathway (Nakamura et al., 2019).

Exposing IECs to microbes induces STAT3 activation; DNA damage; E-cadherin cleavage; and the release of inflammatory cytokines, such as IL-6 and IL-17, which may lead to tumorigenesis (Chung et al., 2018; Liu et al., 2020). Inflammatory diets may lead to obesity and cause inflammation. Inflammatory diets also reduce the release of IL-10, damages the intestinal barrier integrity, increases intestinal permeability, allows the infiltration of bacteria into mucosa, and may cause inflammation. Inflammatory diets initiate the transforming growth factor beta 1 (TGFB1) and NF-kβ signaling pathways and instigate hyperplasia and polyps (Liu L. et al., 2018). TGFB1 is involved in a variety of cellular processes, such as cellular growth, differentiation, and apoptosis. NF-kβ, a nuclear protein, is usually expressed in response to stress, cytokines, and free radicals. **Figure 1** summarizes the interaction among host factors, gut microenvironment, and the microbiota in EOCRC.

## Modulation of GM as Potential Therapeutic Approach in EOCRC

GMs are now considered an effective biotherapeutic agent in the treatment of CRC. Cancer is a primary cause of death worldwide; approximately 9.6 million people died from cancer in 2018 (Nakkarach et al., 2021). Drug resistance in cancer treatment is becoming more common and necessitates an urgent search for a new cancer treatment drug. Gut bacteria, which generate SCFA during fermentation, have been proven to contain anti-cancer properties. SCFA plays an important role in gut homeostasis owing to its anti-cancer, lipid metabolism, anti-inflammatory, and other immune actions.

Management strategies are focused against increasing the severity of CRC by targeting intestinal barrier integrity. Photodynamic therapies using a liposomal formulation of meta-tetra (hydroxyphenyl) chlorin (Reinhard et al., 2015), phytic acid (Liu C. et al., 2018), sildenafil (Islam et al., 2017), alisol B 23-acetate (Zhu et al., 2021), and gegen qinlian decoction (Li et al., 2020b) can reduce inflammation and ameliorate the severity of CRC. Phytic acid and alisol B 23-acetate target MUC2 and tight junction proteins to increase the protective mucosal layer. Trifostigmanoside I, an active compound found in sweet potato (*Ipomoea batata*), can promote the phosphorylation of PKCα/β and ERK1/2 signaling pathways and eventually promote MUC2 production and protect tight junctions (Parveen et al., 2021).

VSL#3 is a probiotic that contains Lactobacillus species (L. acidophilus, L. bulgaricus, L. paracasei, L. plantarum), Bifidobacterium species (B. breve, B. infantum, B. longum) and Streptococcus species (S. thermophilus) (Kumar et al., 2017). Lactobacillus species secrete lactosepins, which degrades IFN-γ-induced protein 10 and subsequently leads to the attenuation of inflammatory responses. Lactobacillus and Bifidobacterium species secrete linoleic acid, which can reduce the accumulation of macrophages and peroxisome proliferator-activated receptor-γ and consequently reduce inflammation. The effectiveness of probiotic mixture VSL#3 was proven although DSS-induced colitis mice were used, which were MUC2 deficient (Kumar et al., 2017). The beneficial effect of VSL#3 is exerted through the modification of growth factors; the prevention of the loss of tight junction proteins, occludin and zonula occludens 1; the increased production of antimicrobial peptides, Ang4 and Reg3β; and the increased production of SCFAs. The colonic permeability of mice was reduced by acetate and proprionate (SCFAs), which further reduced the microbial influx.

A systematic review of 23 randomized clinical trials unveiled numerous benefits of probiotic supplementation in CRC such as increased in GM diversity, reduction in post-operative infection complications, inhibition of pro-inflammatory cytokine production, improved side effects of chemotherapy and improved surgery success rate (Dikeocha et al., 2021). Some of the bacteria of interest includes *Lactobacillus* (*L. uteri*, *L. helveticus*, *L. bulgaricus*, *L. casei*), *Bifidobacterium* (*B. lactis*, *B. breve*), *Saccharomyces boulardii*, *Bacillus polyfermenticus*, *Butyrivibrio fibrisolvens* and *Propionibacterium* species (Uccello et al., 2012; Dikeocha et al., 2021). A study consisting of 77 samples of CRC, polyps and healthy feces by Zinatizadeh et al. (2018) revealed significant difference in average copy number of *Lactobacillus acidophilus* between polyps and CRC groups, compared to healthy groups (p<0.0001).

GM modulation as CRC management strategies is currently being explored. However, researchers have yet to discover definitive criteria of healthy GM, or established standard protocols for employing GM as medical treatment or management of CRC.

## Conclusion

The incidence of EOCRC has risen at an alarming rate in recent decades and has become a global problem. Unhealthy diet and physical inactivity negatively affect microbiota diversity and the overall physiological homeostasis in the gut. Our review provides an insight into the possible underlying mechanism of EOCRC through dysbiosis. The roles of *F. nucleatum*, *B. fragilis* and *E. coli* in initiating cancer are intensified with obesity, inflammatory diet, heme intake, mutagens, and stress. Individuals’ exposure to insults from micro- and macroenvironment start from before birth. Future studies may focus on exposome influence on microbiota diversity in relation to health.

## Author Contributions

RR and NM drafted the outline and in charge of the original ideas of the project. SA, NM, KN, and HA drafted the manuscript. NM and RR revised and edited the manuscript. All authors contributed to the article and approved the submitted version.

## Funding

The research was funded by the Research University fund, code DIP-2020-014.

## Conflict of Interest

The authors declare that the research was conducted in the absence of any commercial or financial relationships that could be construed as a potential conflict of interest.

## Publisher’s Note

All claims expressed in this article are solely those of the authors and do not necessarily represent those of their affiliated organizations, or those of the publisher, the editors and the reviewers. Any product that may be evaluated in this article, or claim that may be made by its manufacturer, is not guaranteed or endorsed by the publisher.
